# Using mitochondrial respiration inhibitors to design a novel model of bipolar disorder-like phenotype with construct, face and predictive validity

**DOI:** 10.1038/s41398-021-01215-y

**Published:** 2021-02-12

**Authors:** O. Damri, S. Asslih, N. Shemesh, S. Natour, O. Noori, A. Daraushe, H. Einat, N. Kara, G. Las, G. Agam

**Affiliations:** 1 Department of Clinical Biochemistry and Pharmacology and Psychiatry Research Unit, Faculty of Health Sciences, Ben-Gurion University of the Negev and Mental Health Center, Beer-Sheva, Israel; 2 School of Behavioral Sciences, Tel Aviv-Yaffo Academic College, Tel Aviv-Yafo, Israel

**Keywords:** Bipolar disorder, Molecular neuroscience

## Abstract

We mimicked mild mitochondrial-distress robustly reported in bipolar-disorder (BD) by chronic exposure to uniquely low doses of inhibitors of mitochondrial-respiration complexes in vitro and in vivo. Exposure of the neuronal-originating SH-SY5Y cells to very low dose (10 pM) rotenone, a mitochondrial-respiration complex (Co)I inhibitor, for 72 or 96 h did not affect cell viability and reactive oxygen species (ROS) levels. Yet, it induced a dual effect on mitochondrial-respiration: overshooting statistically significant several-fold increase of most oxygen-consumption-rate (OCR) parameters vs. significantly decreased all OCR parameters, respectively. Chronic low doses of 3-nitropropionic acid (3-NP) (CoII inhibitor) did not induce long-lasting changes in the cells’ mitochondria-related parameters. Intraperitoneal administration of 0.75 mg/kg/day rotenone to male mice for 4 or 8 weeks did not affect spontaneous and motor activity, caused behaviors associated with mania and depression following 4 and 8 weeks, respectively, accompanied by relevant changes in mitochondrial basal OCR and in levels of mitochondrial-respiration proteins. Our model is among the very few BD-like animal models exhibiting construct (mild mitochondrial dysfunction), face (decreased/increased immobility time in the forced-swim test, increased/decreased consumption of sweet solution, increased/decreased time spent in the open arms of the elevated plus maze) and predictive (reversal of rotenone-induced behavioral changes by lithium treatment) validity. Our rotenone regime, employing doses that, to the best of our knowledge, have never been used before, differs from those inducing Parkinson’s-like models by not affecting ROS-levels and cell-viability in vitro nor motor activity in vivo.

## Introduction

Mitochondrial dysfunction (MD) has become a well-established facet of the pathophysiology of neuropsychiatric [for reviews see refs. ^1,2^]. Findings include disturbances in mitochondrial enzymes, calcium signaling, and energy metabolism^2,3^, corroborating with the postmitotic nature of neurons, their utmost metabolic dependence on mitochondria^4^. Reports in bipolar-disorder (BD) comprise downregulated expression of mitochondria-related genes and proteins, an association of mtDNA mutations with the disorder and decreased complex I (CoI) subunits’ protein levels^3^. MD in BD is also reflected in impaired mitochondrial network organization and localization^5^. Magnetic-resonance spectroscopy (MRS) and two-dimensional proton echo-planar spectroscopic imaging (PEPSI) show impaired electron-transport-chain (ETC)/oxidative-phosphorylation system (OxPhos), decreased energy production and a shift towards glucose metabolism^6^. Brain metabolic impairment appears more prominent in BD-I than in BD-II, suggesting a correlation between the severity of MD and the severity of the illness^7^. Moreover, bipolar patients often exhibit symptoms characterizing subjects with mitochondrial disorders^8^. Corroborating with these studies numerous reports demonstrate beneficial effects of mood-stabilizers on mitochondrial-function (MF)^9–12^. We have previously shown that treating mice with lithium upregulates MF-related genes in the frontal-cortex and the hippocampus^13^.

To circumvent the limitation of the lack of an animal model that presents both the depressive and the manic poles of BD, the present study aimed to establish models that recapitulate mild MD in vitro (in neuronal cells in culture) and in vivo (in male mice). We employed rotenone and 3-nitropropionic acid (3-NP), mitochondrial CoI and CoII inhibitors, respectively, widely used in modeling central facets of the neurodegenerative disorders Parkinson’s disease (PD) and Huntington’s disease (HD), respectively. Rotenone causes an extreme alteration in mitochondrial homeostasis and blocks autophagic flux by promoting an increase in ROS (O_2_•− in particular) and by impairing lysosomal integrity^14^. Sources and mechanisms suggested for the generation of ROS include dopamine metabolism, overexpression of α-synuclein, neuroinflammatory cells and MD^15^. 3-NP inhibits mitochondrial CoII irreversibly and crosses the blood–brain-barrier^16^. 3-NP treated rodents present aberrant gait [decreased stride length (the distance between two successive hind limb prints), increased footprint length (the distance from the heel to the tip of the third digit of the hind limb), and lack of the superimposition of the footprints of the hind and forelimbs observed in control animals^17^], inability to balance over a narrow beam, deficits in foraging or exploratory behaviors, and increased anxiety and/or depression^18^.

In relation to the in vitro model, we hypothesized that: I. Neuronal cells cope with very mild respiratory distress induced by exposure to very low rotenone or 3-NP doses for a relatively short duration; II. Mild respiratory distress induced by chronic exposure to low rotenone or 3-NP doses does not result in apoptosis, but mitochondrial fragmentation does occur leading to neuronal dysfunction and psychiatric-like characteristics (e.g., BD). Pertaining to the in vivo model we hypothesized that mild respiratory distress induced by chronic exposure to low rotenone doses induces duration-dependent BD-like behavior partially or totally reversed by lithium treatment. Accordingly, here we show that exposure of human neuronal cells (SH-SY5Y) in vitro, and male mice, in vivo, to low rotenone doses (2.5-fold lower than the lowest used to induce PD-like models and, to the best of our knowledge, never used before^19^) results in mania- and depression-associated characteristics in a doseXtime-dependent manner. Unlike the results with rotenone, chronic exposure of human neuronal cells to low 3-NP concentrations (at least five-fold lower than those used to induce HD-like characteristics and rarely used before^20^) resulted in reduced cell viability (CV).

## Materials and methods

### Cell culture

Human neuroblastoma cells, SH-SY5Y (ATCC, Manassas, VA), were maintained in DMEM medium (Biological Industries, Beit-Haemek, Israel) supplemented with 1% fetal bovine serum (FBS, Biological Industries, ibid) as previously described^21^. Cultures were grown for 24 h, then the medium was changed to that containing rotenone (10, 10^2^, 10^3^, 10^4^ pM) or 3-NP (1, 10, 100, 1000 nM) dissolved in DMSO (Sigma-Aldrich, St. Louis, MO) or vehicle for 6–96 h.

### Animals

Male, 5- or 7-week-old Institute of Cancer Research (ICR) male mice (Envigo, Nes-Ziona, Israel) were maintained on a 12 h/12 h light/dark cycle (lights on between 7:00 a.m. and 7:00 p.m.) with the constant temperature at 23 ± 1 ^o^C and ad libitum access to food and water. All tests were performed during the light phase of the cycle between 9:00 am and 7:00 pm. The mice were allowed to acclimatize to the new environment for one week before treatment initiation. Animals were weighed once every third day and their general well-being assessed by examination of their fur and general appearance. Neither of the treatments induced any discerned health problems. Since all mice for any given experiment were obtained on the same day from Envigo, randomization among the experimental groups was not required. All procedures followed Israeli guidelines for treatment and care of experimental animals and were approved by the Ben-Gurion University animal experimentation ethics committee (Authorization Number: IL-50-07-2015). Rotenone (Sigma-Aldrich, ibid) was dissolved in saline supplemented with 0.5% DMSO (ibid) and injected subcutaneously (s.c.) at 0.25, 0.5, 0.75, 1.25, and 1.5 mg/kg once daily for 2, 4 or 8 weeks. Control mice received vehicle (saline supplemented with 0.5% DMSO). D-Amphetamine was injected as described^13,22^. For lithium administration regime see Supplementary material, Methods. All drugs were injected daily at the same time of the day including the days of the behavioral tests.

### Cell viability

CV was determined by the MTT (3-(4,5-dimethylthiazol-2-yl)-2,5-diphenyltetrazolium bromide) assay (Sigma, St. Louis, MO) according to the manufacturer’s instructions.

### ApoLive-Glo multiplex assay for CV and apoptosis

Since we used rotenone or 3-NP, affecting mitochondrial-respiration, we chose to confirm the results obtained using the MTT assay using a method not based on mitochondrial activity. To this end we used the ApoLive-Glo multiplex assay kit (Promega, Madison, WI) according to the manufacturer’s instructions. The activity of a protease marker monitors CV and apoptosis is measured based on caspase-3/7 activation.

### Mitochondrial parameters—mass, membrane-potential, and ROS-levels

The Operetta High-Content Image System (Perkin-Elmer, Waltham, MA) was used to assess cells’ mitochondrial-mass (MM), mitochondrial-membrane-potential (MMP/ΔψM) and superoxide levels (reflecting ROS-levels) utilizing the molecular probes mitotracker-green (MTG, 100 nM, Molecular Probes, Eugene, OR), tetramethyl-rhodamine ethyl ester (TMRE, 50 nM, Invitrogen, Camarillo, CA) and mitochondrial superoxide indicator (MitoSOX red, 2.5 nM, Invitrogen, ibid), respectively. Nuclear staining by Hoechst 33258 dye (40 nM, Sigma, ibid) was used to identify cells’ location. The software’s output is the average intensity of MTG/TMRE/MitoSOX per cell based on Hoechst staining. ΔψM values were normalized to MM^23^. MitoSOX intensity was collected only when co-localized with MTG and was further normalized to ΔψM^24^. This normalization was done since it has been shown that the MitoSox color is sensitive to pH^24^.

### Mitochondrial-respiration parameters

Oxygen consumption rate (OCR) was measured using the Seahorse XF-24 Extracellular Flux Analyzer (Seahorse Biosciences, North Billerica, MA) according to the manufacturer’s instructions. For the SH-SY5Y cells the procedure required seeding the cells the day prior to the experiment—8 × 10^3^ cells/well for 96 h experiments, 50 × 10^3^ cells/well for acute experiments, in 600 μl growth medium in XF plates (Agilent Technologies, Santa Clara, CA) or brain - specimens dissected on dry ice were weighed, cut into small pieced, placed on a net in Seahorse XFe24 Islet Capture FluxPak plates (Seahorse Biosciences, ibid). Each sample was assayed in triplicate. OCR parameters were calculated using Seahorse Bioscience software (version 3.3). Results were normalized to protein concentration in each well.

### Protein concentration

Protein was extracted from SH-SY5Y cells after harvesting from the plate or from the dissected brains (stored at −80 ^0^C until further handling) by sonication for 10 s at 4^o^C and 50% power capacity (Heat System Ultasonic, Newtown, CT) in RIPA lysis buffer. Protein concentration was determined using NanoDrop 2000 (Thermo Scientific, Waltham, MA).

### ATP levels and mitochondrial OXPHOS complex I enzymatic activity

ATP in lyzed cells and CoI enzymatic activity were measured using dedicated kits (Abcam, Cambridge, UK) according to the manufacturer’s recommendations.

### Western blotting

Western blotting was performed according to a standard protocol used in our laboratory^25^, each sample tested in duplicate of 10 and 20 µg/lane, to verify linearity. For antibodies details see Supplementary Table I. Results were normalized to Ponceau staining (total protein^26^, Sigma-Aldrich, ibid).

### Behavioral tests

Tests representing domains of BD were used to evaluate the outcome caused by induction of mild mitochondria dysfunction^22^. Each test was run on a separate day. The order of the battery of the tests was from the least to the most intrusive to limit the effect of previous tests on the behavior of the following tests. The order of the tests was as follows^27^: (1) Open field. (2) Rotarod. (3) Elevated plus-maze (EPM). (4) Sweet-solution preference (SSPT). (5) Social-interaction. (6) Forced-swim (FST). (7) Amphetamine-induced hyperlocomotion. All tests were carried out as previously elaborated^22^. The investigator who ran the tests was blinded to the group allocation during the experiment as well as when assessing the outcome.

### Statistical analysis

Results are given as means ± SEM either of the original values or of the % of the mean of the control. The latter normalization was carried out for experiments preformed several times at different time points and/or using different batches of kits/plates. Results exceeding ±2SDs were excluded. A minimal number of replicates/animals per group were according our lab’s years of experience. Statistical analysis was carried out by two- or three-way ANOVA followed by Fisher’s Least Significant Difference (LSD) post hoc test or by repeated-measures ANOVA followed by post hoc LSD test, as appropriate and as indicated for each analysis, using STATISTICA version 13 (StatSoft, Tulsa, Oklahoma). *p* ≤ 0.05 was considered statistically significant. All data met the assumptions of normal distribution within a given group and within groups and the variance between the groups that were statistically compared was similar.

Data from the Seahorse XF Apparatus were exported to Excel 2010 for further analysis. The serial nature of the measurements obtained using the apparatus’ software allowed the use of repeated-measures ANOVA for some of the experiments.

## Results

### Allocating a regime of mild MD in SH-SY5Y cells

**Fig. 1:**
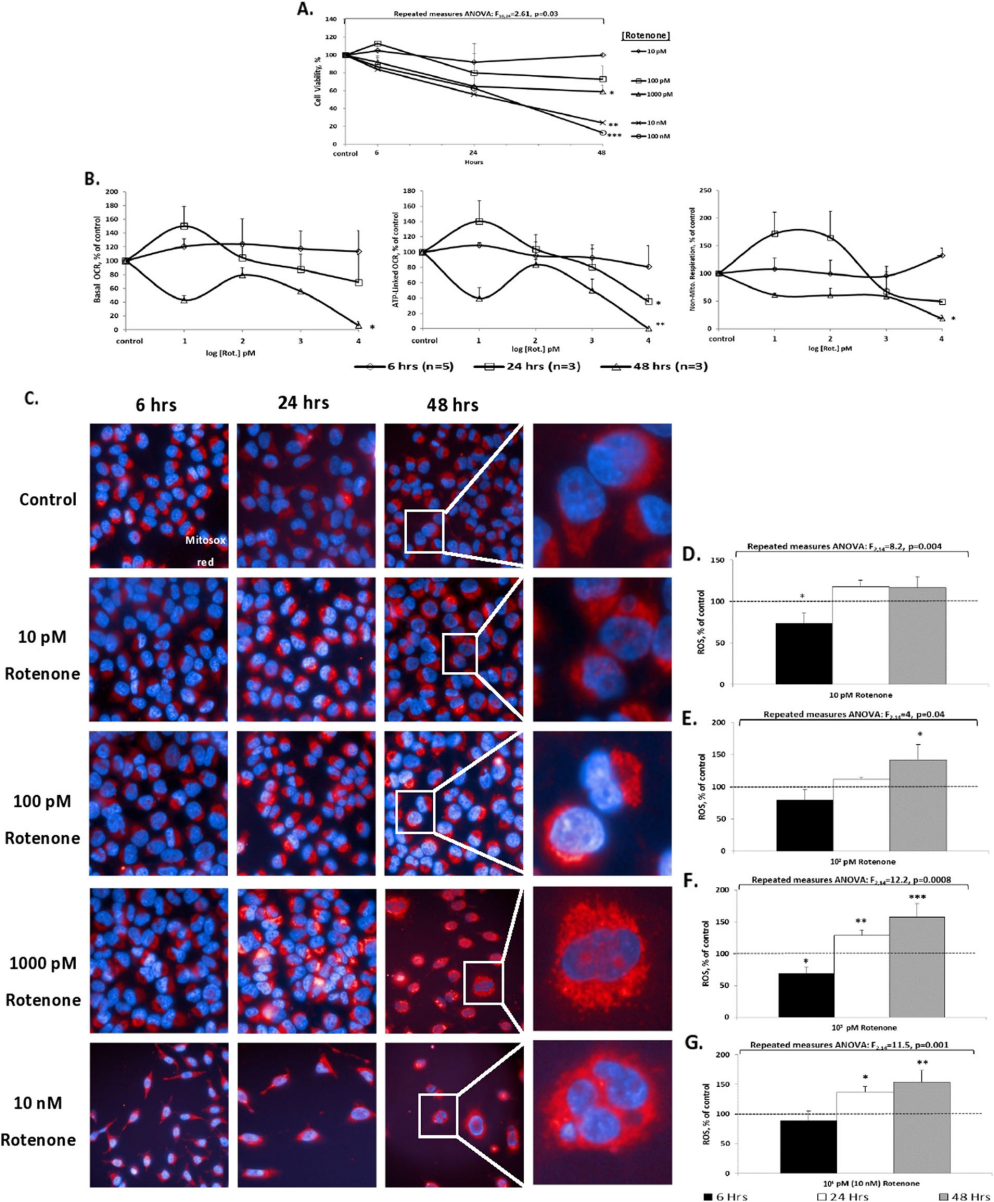
Dose-response and duration-dependence of the effect of rarely used before (10 pM–10 nM) rotenone concentrations on cellular and mitochondrial parameters. Results expressed in percentage of control (vehicle-treated) represent means ± SEM of three independent experiments, each in triplicate. **A** Cell viability (MTT assay). Control value was: 0.336 ± 0.034 OD_540 nm_. Fisher’s LSD post hoc test: *1000 pM rotenone differed significantly from the control: *p* < 0.02 for 24 and *p* < 0.007 for 48; **10 nM rotenone: *p* < 0.004 for 24 h and *p* < 5E^−06^ for 48 h; ***100 nM rotenone: *p* < 0.02 for 24 h and *p* < 4E^-07^ for 48 h. **B** Mitochondrial respiration parameters. Results in pmoles O_2_ consumed/min/mg protein converted into % of control. Control values: basal OCR – 2.3 ± 0.8; ATP-linked OCR – 2.08 ± 1.1; non-mitochondrial respiration – 1.85 ± 1.16. One result exceeding ±2SDs was excluded. Basal OCR: *48 h, ANOVA: *F*_4,7_ = 14.2, *p* = 0.001; post hoc Fisher’s LSD test: 10, 10^3^, and 10^4^ pM rotenone (Rot.) vs*.* control, *p* < 0.02. ATP-linked OCR: *24 h, ANOVA: *F*_4,10_ = 5.1, *p* = 0.01; post hoc Fisher’s LSD test: 10^4^ pM (Rot.) vs*.* control, 10 and 10^2^ pM, *p* < 0.03. **48 h, ANOVA: *F*_4,9_ = 8.9, *p* = 0.003; post hoc Fisher’s LSD test: 10, 10^3^, and 10^4^ pM (Rot.) vs*.* control, *p* < 0.03. Non-Mitochondrial respiration: *48 h, ANOVA: *F*_4,8_ = 6.7, *p* = 0.01; post hoc Fisher’s LSD test: all (Rot.) concentrations vs*.* control, *p* < 0.04; 10^4^ pM rotenone vs*.* 10 and 10^2^ pM, *p* < 0.05. **C**–**G.** ROS levels. Results of fluorescence intensity, (>30 cells analyzed/well) ± SEM. Control ROS values were 555 ± 59 a.u. A gradual increase in ROS levels in a dose-response and time-dependent manner was observed, presented in representative images (Hoechst and mitosox staining - **C**) and bar-graphs (**D**–**G**). **D** 10 pM rotenone: Fisher’s LSD post hoc test: *6 h, a significant decrease in ROS levels vs*.* all treatments, *p* ≤ 0.005. **E** 10^2^ pM rotenone: Fisher’s LSD post hoc test: *48 h, a significant increase in ROS levels vs*.* control and 6 h, *p* < 0.03. **F** 10^3^ pM rotenone: Fisher’s LSD post hoc test: *6 h, a significant decrease in ROS levels vs*.* all treatments, *p* < 0.02; **, ***24 and 48 h, respectively, a significant increase in ROS levels vs*.* control and 6 h, *p* < 0.02. **G** 10^4^ pM (10 nM) rotenone: Fisher’s LSD post hoc test: *, **24 and 48, respectively, a significant increase in ROS levels vs. control and 6 h, *p* < 0.03.

To possibly model psychiatric-like (e.g., bipolar) rather than neurodegenerative (e.g. Parkinson’s)-like disorders SH-SY5Y cells were exposed to a range of concentrations (including low concentrations rarely used before) of two mitochondrial-respiration inhibitors—rotenone, 10–10^5^ pM for 6–48 h (Fig. 1 and Supplementary Fig. 1A, B) and 3-NP, 1–10^3^ nM for 6–48 h (Supplementary Fig. 1C).

As hypothesized, CV (Fig. 1A and Supplementary Fig 1 Ci) was not significantly affected neither by 10 and 10^2^ pM rotenone nor by 1 nM 3-NP following exposure for up to 48 h. Exposing the cells to either 10^3^–10^5^ pM rotenone or 10^2^–10^3^ nM 3-NP for ≥24 h did result in a significant reduction. When rotenone’s effect on CV was measured based on live-cell protease activity (Supplementary Fig. 1A), 6 h of exposure to 10^3^–10^5^ pM resulted in a significant reduction, providing credence to the trend of reduced CV obtained under these conditions using the MTT assay (Fig. 1A). Apoptosis was discerned only following exposure to 10^5^ pM rotenone for 24 h (Supplementary Fig. 1A), in accordance with the reduction in CV.

By-and-large, rotenone treatment induced a gradual duration- and dose-dependent reduction in the respiration parameters mitochondrial basal- and ATP-linked OCR and non-mitochondrial-respiration (Fig. 1B) except for the 24 and 48 h of 10 pM. Exposure to 10^4^ pM (10 nM) rotenone for 48 h reduced all parameters, apparently since most of the cells did not survive (compatible with the CV results). ROS-levels exhibited a semi-gradual significant increase in a dose- and duration-dependent manner. Namely, 10 pM rotenone did not affect the levels, 10^2^ pM significantly increased them following 48 h of exposure, 10^3^ and 10^4^ pM—significantly increased the levels following 24 and 48 h (Fig. 1C–G). In parallel, exposure to 10^4^ pM for 48 h significantly decreased MMP with a trend towards decrease observed already following 24 h (Supplementary Fig. 1Bi, Bii). Exposure to 10^4^ pM for 24 and 48 h significantly increased MM (Supplementary Fig. 1Bi, Biii), possibly reflecting an attempt to compensate for the mitochondrial insult. Furthermore, exposure to these relatively high concentrations induced nuclei fragmentation (Fig. 1C, enlarged frames), compatible with the CV results.

**Fig. 2:**
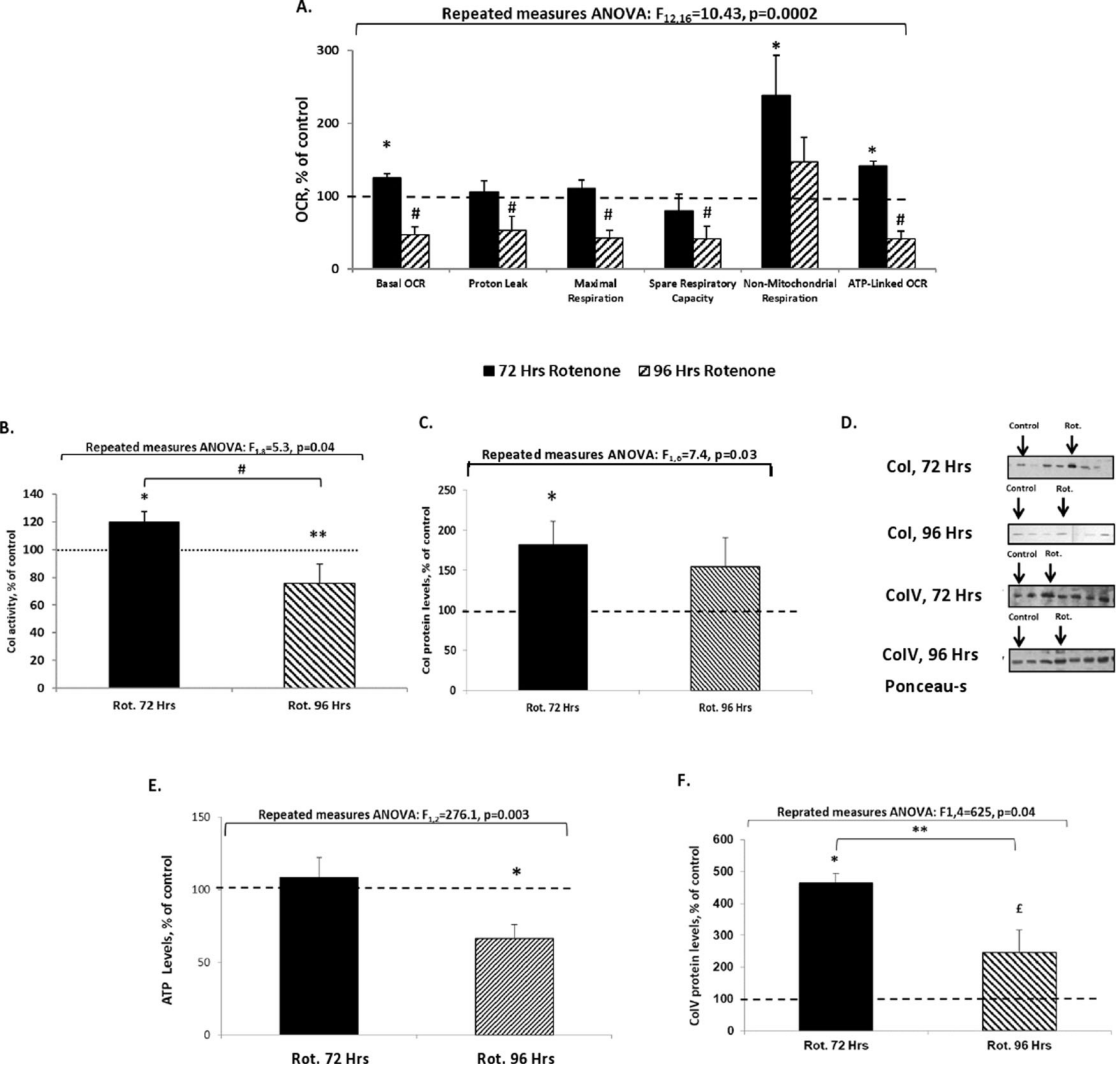
Mitochondrial function-related parameters following exposure to 10 pM rotenone for 72 and for 96 h. Results expressed in percentage of control (vehicle-treated) represent means ± SEM of at least four independent experiments, each in triplicate. **A** Respiration parameters. Results in pmoles O_2_ consumed/min/mg protein (converted into % of control) of four independent experiments, each point in duplicate. Control values were: Basal OCR – 8.35 ± 2.02; Proton leak – 1.79 ± 0.25; Maximal respiration – 18.04 ± 6.90; Spare capacity – 9.70 ± 2.85; Non-mitochondrial OCR – 2.19 ± 1.57; ATP-linked OCR – 6.55 ± 1.77. Two results exceeding ±2SDs were excluded. Fisher’s LSD post hoc test revealed that exposure to 10 pM rotenone for 72 h resulted in significant upregulation of basal OCR, non-mitochondrial respiration, and ATP-linked OCR, *p* < 0.05, while exposure to this concentration for 96 h led to a significant downregulation of all respiration parameters except for non-mitochondrial OCR, *p* < 0.04. **B**–**D** Complex I activity and protein levels. Results of five independent experiments, each in triplicate. **B** Complex I activity. Control value was: 98 ± 39 OD_540 nm_. Exposure to 10 pM rotenone (Rot.) significantly enhanced and decreased CoI activity following 72 and 96 h, respectively. Fisher’s LSD post hoc test: ^#^72 h vs*.* 96 h of exposure, *p* = 0.01; *one-tailed post hoc Fisher’s LSD test: 72 h vs*.* control, *p* = 0.05; **one-tailed post hoc Fisher’s LSD test: 96 h vs*.* control, *p* = 0.02. **C** Complex I protein levels. Control value was: 2.8 ± 0.6 arbitrary units (a.u.). Fisher’s LSD post hoc test: *exposure for 72 h demonstrated a significant increase vs. control, *p* = 0.02. **D** Representative blots of CoI protein bands and Ponceau-S (total protein, for normalization). **E** ATP levels. Results of four independent experiments, each in triplicate, obtained in a.u. and converted into % of control. Control value was: 7.2 ± 2.2 a.u. Exposure to 10 pM Rot. for 96 h significantly decreased ATP levels. Fisher’s LSD post hoc test: *96 h vs*.* control, *p* < 0.05. **F** Complex IV protein levels. Results of five independent experiments, each in triplicate. The control value was: 4.2 ± 1.6 a.u. Exposure to 10 pM Rot. for 72 h and for 96 h significantly increased complex IV protein levels. Fisher’s LSD post hoc test: *72 h Rot. vs*.* control, *p* = 0.00001; ^£^96 h Rot. vs. control, *p* = 0.001; **72 vs. 96 h of Rot. vs. control, *p* = 0.00001.

The results using 3-NP (Supplementary Fig 1Ci–Civ) demonstrated a different pattern. For details see Supplementary information, Results. This is not surprising since unlike Col and CoIII, CoII is not the main complex responsible for ROS production^28^. Because we aimed to implement a useful tool to model a robust chronic mild MD as reported in BD we proceeded in assessing additional mitochondrial parameters only following exposure to rotenone (10 pM) for 72 and 96 h. Contrarily to the rotenone-induced Parkinson’s-like model, and in agreement with our hypothesis, exposure of SH-SY5Y cells to this very low concentration for 72 or 96 h did not affect apoptosis, CV, MM, MMP, and ROS-levels (data not shown) but did result in an intriguing mode of alterations in mitochondrial-respiration-related parameters. Exposure for 72 h significantly increased basal and ATP-linked OCR and non-mitochondrial respiration (Fig. 2A), possibly in an attempt to overcome their significant attenuation following the exposure for 48 h (Fig. 1B). Intriguingly, 96 h of exposure significantly reduced basal and ATP-linked OCR (Fig. 2A). Along with the results of basal and ATP-linked OCR and non-mitochondrial respiration following 24 and 48 h of exposure to 10 pM (Fig. 1B)—a pattern of fluctuations reminiscent of the extreme mood swings characterizing BD is discerned. The increase in basal and ATP-linked OCR corroborated with the significant increase in CoI activity and protein levels (Fig. 2B–D). Exposure for 96 h significantly attenuated CoI activity (Fig. 2B), possibly responsible for the reduction in all mitochondrial OCR parameters and ATP levels (Fig. 2A, E). CoIV protein levels, indicative of mitochondrial abundance^29^, were significantly increased following exposure for both 72 and 96 h (Fig. 2F), albeit, to a significantly lesser extent following 96 h. Unlike in the Parkinson’s-like model, our regime did not increase ROS-levels nor decreased CV, but did affect most OCR parameters, mimicking the state in BD^30^.

### Behavioral consequences associated with BD of in vivo rotenone-induced mild MD in male mice and their reversal by lithium treatment

**Fig. 3:**
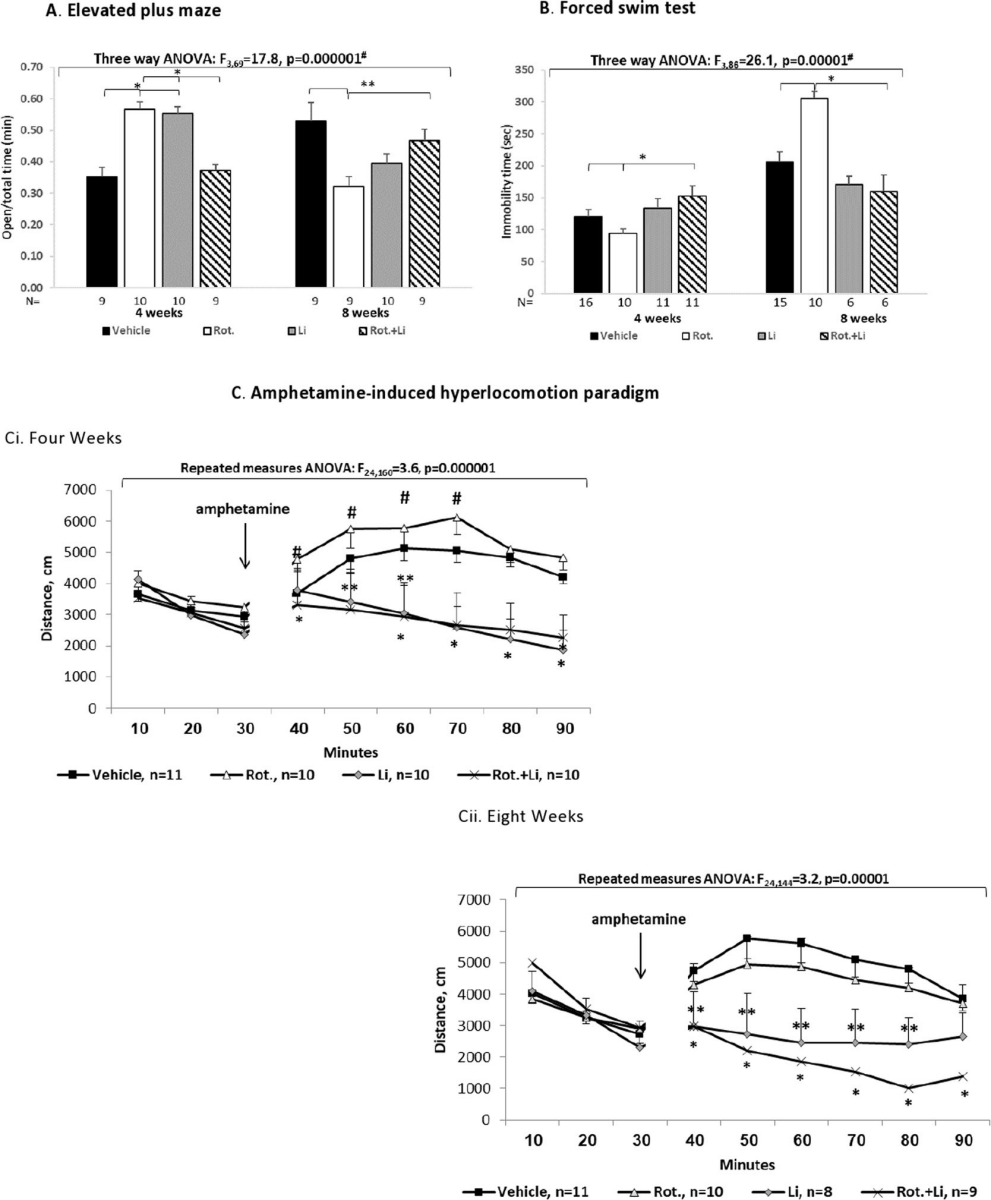
Behavioral consequences of in vivo rotenone-induced mild mitochondrial dysfunction in mice and their reversal by lithium treatment. 0.75 mg/kg/day rotenone (Rot.) was administered i.p. for 4 or 8 weeks. Lithium (Li) was given for the last 2 weeks of rotenone treatment. Results represent means ± SEM of three independent experiments with at least three mice/group in each experiment. In each test (EPM, FST and amphetamine-induced hyperlocomotion) one result exceeding ±2SDs was excluded. **A** The effect on the behavior in the EPM. Time in the open arms/total time was increased by Rot. following 4 weeks and reduced following 8 weeks of treatment. Three-way ANOVA with treatment (± Rot.), Li (±) and duration of treatment (4/8 weeks) as main factors: ^#^values are of the TreatmentXLiXDuration interaction; *Fisher’s LSD post hoc test: 4/8 weeks Rot. or Li vs. vehicle and vs. Rot.+Li, *p* < 0.04. **B** The effect on the behavior (immobility time) in the FST. Three-way ANOVA with treatment (± Rot.), Li (±) and duration of treatment (4/8 weeks) as main factors: ^#^values are of the TreatmentXLiXDuration interaction; *Fisher’s LSD post hoc test: 4/8 weeks Rot. vs. vehicle and vs. Rot.+Li, *p* < 0.03. **C** The effect in the amphetamine-induced hyperlocomotion paradigm. Ci. Repeated measures ANOVA of the results following amphetamine injection revealed that Rot. significantly augmented the effect of amphetamine (^#^Fisher’s LSD post hoc test, *p* < 0.03). Li by itself significantly reversed amphetamine’s effect (**p ≤ 0.03) and totally reversed the effect of Rot. on amphetamine-induced hyperlocomotion (*p < 0.003). Cii. Repeated measures ANOVA of the results following amphetamine treatment revealed that Li by itself significantly reversed amphetamine’s effect (**p ≤ 0.05) and totally abolished amphetamine-induced hyperlocomotion (*p < 0.0001).

**Fig. 4:**
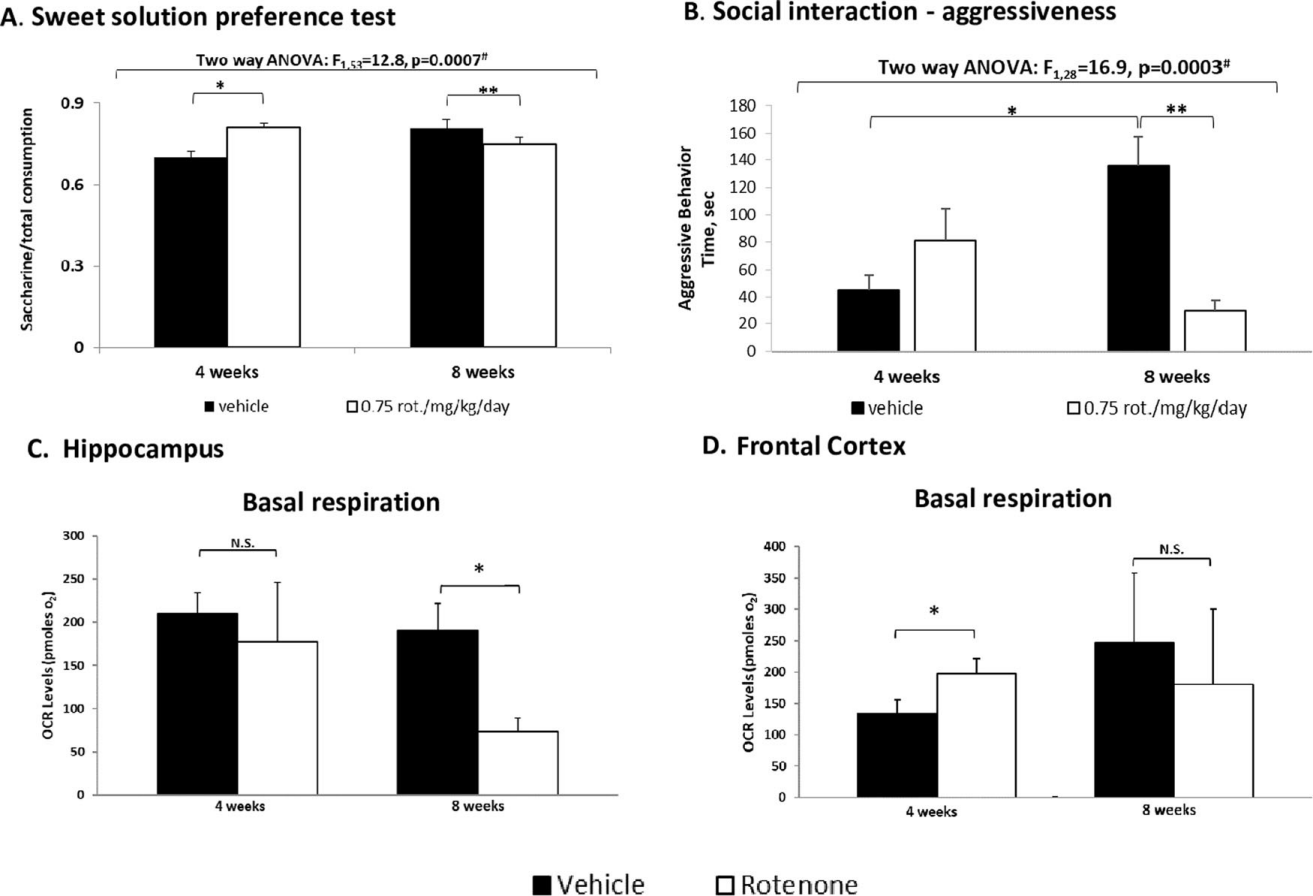
Sweet solution preference test, aggressive behavior, and brain mitochondrial basal respiration of mice following in vivo rotenone-induced mild MD. Rotenone (0.75 mg/kg/day) was administered i.p. for 4 or 8 weeks. Lithium (Li) was given for the last 2 weeks of rotenone treatment. Results represent means±SEM of 12 mice/group. One result of the SSPT and one result from the basal respiration data which exceeded ±2SDs were excluded. **A** The effect in the SSPT. The amount of saccharine/total fluids drank was significantly increased following 4 weeks of treatment and significantly decreased following 8 weeks. Two-way ANOVA with treatment (±Rot.) and duration of treatment (4/8 weeks) as main factors: ^#^values are of the TreatmentXDuration interaction; Fisher’s LSD post hoc test: *4/8 weeks of Rot. vs. vehicle, *p* < 0.02. **B** The effect on aggressiveness. Time spent in aggressive behavior was 1.8-fold non-significantly increased following 4 weeks of treatment and 4.6-fold significantly decreased following 8 weeks. Two-way ANOVA with treatment (±Rot.) and duration of treatment (4/8 weeks) as main factors: ^#^values are of the TreatmentXDuration interaction. Fisher’s LSD post hoc test: *8 weeks of vehicle vs. 4 weeks of vehicle, *p* < 0.0009; **8 weeks rotenone vs. vehicle, *p* = 0.0001. **C** The effect on hippocampal mitochondrial basal respiration. *t-test analysis showed a significant reduction following 8 weeks of Rot. (*p* = 0.01). **D** The effect on frontal-cortex mitochondrial basal respiration. *t-test analysis showed a significant increase following 4 weeks of Rot. (*p* = 0.05).

In an attempt to establish an in vivo model of mild MD adult male were administered very low, rarely used, rotenone doses (0.25–1.5 mg/kg/day) for 2, 4, and 8 weeks followed by a set of behavioral tests (Supplementary Fig. 2A–C). As anticipated, unlike in the Parkinson’s-like model, neither of the rotenone regimes tested affected spontaneous or motor activity (Supplementary Fig. 2A, B). Yet, 0.75 and 1.5 mg/kg/day rotenone did induce affective-like behavioral changes (Figs. 3, 4 and Supplementary Fig. 2C). 1.5 mg/kg/day induced an increase in immobility in the FST following 4 and 8 weeks of treatment (ref. ^31^ and Supplementary Fig. 2C). The results summarized in supplementary Fig. 2 led us to choose the 0.75 mg/kg/day for 4 and 8 weeks for the further experiments. 0.75 mg/kg/day induced affective-like behavioral changes in a duration-dependent manner: 4 weeks induced manic-like characteristics of prolonged exposure to the less safe arms in the EPM (Fig. 3A), hedonic-like behavior reflected in significantly increased consumption of saccharine in the SSPT (Fig. 4A)—an anti-depression-/mania-associated phenotype (based on the animals’ natural preference for sweets^32^), compatible with the reduced immobility (hyperactivity) in the FST (Fig. 3B and Supplementary Fig. 2C). Eight weeks of administration of the same dose resulted in significantly reduced time spent in the open arms of the EPM (Fig. 3A), significantly longer immobility time in the FST (Fig. 3B and Supplementary Fig. 2C) and reduced saccharine consumption in the SSPT (ref. ^31^ Fig. 4A). The social-interaction test results were also duration-dependent in a dichotomic manner. Four weeks-injected male mice (vehicle or rotenone)—significantly more social/less aggressive behavior compared with 8 weeks vehicle-treated mice that were highly aggressive, an effect significantly attenuated by rotenone (Fig. 4B). Long-term injections might elevate stress^33^ and, consequently, increase aggressive behavior^34^. In the amphetamine-induced hyperlocomotion paradigm 4 weeks of rotenone significantly enhanced amphetamine-induced mania-associated effect (Fig. 3Ci) corroborating our lab’s previous finding^13^. Eight weeks of rotenone treatment did not affect the response to amphetamine (Fig. 3Cii).

We assessed the predictive validity of our model in three representative tests—EPM, FST and amphetamine-induced hyperlocomotion—by treating the mice with lithium during the last 2 weeks of the exposure to rotenone. Interestingly, in the EPM, lithium by itself affected the behavior in a similar manner to rotenone only, but, as hypothesized, there was a statistically significant interaction between the effects of rotenone and lithium which indicated that lithium reversed the effect of rotenone following 4 and 8 weeks of treatment (Fig. 3A). In the FST, unlike in the EPM, lithium by itself did not affect the behavior, but, as hypothesized, there was a statistically significant interaction between the effects of rotenone and lithium which indicated that lithium reversed the mania- and depression-associated effects of rotenone (Fig. 3B). In the amphetamine-induced hyperlocomotion, as expected, amphetamine enhanced the distance traveled by the mice, rotenone had a dual effect—significantly augmented the effect of amphetamine following 4 weeks of treatment (Fig. 3Ci) and significantly attenuated amphetamine’s effect following 8 weeks of treatment (Fig. 3Cii). Lithium by itself significantly reversed amphetamine’s effect both following 4 and 8 weeks of rotenone treatment and also totally reversed the effect of 4 and 8 weeks of rotenone. For detailed statistical analyses of the effects of rotenone and lithium please see Fig. 3 and its legend.

**Fig. 5:**
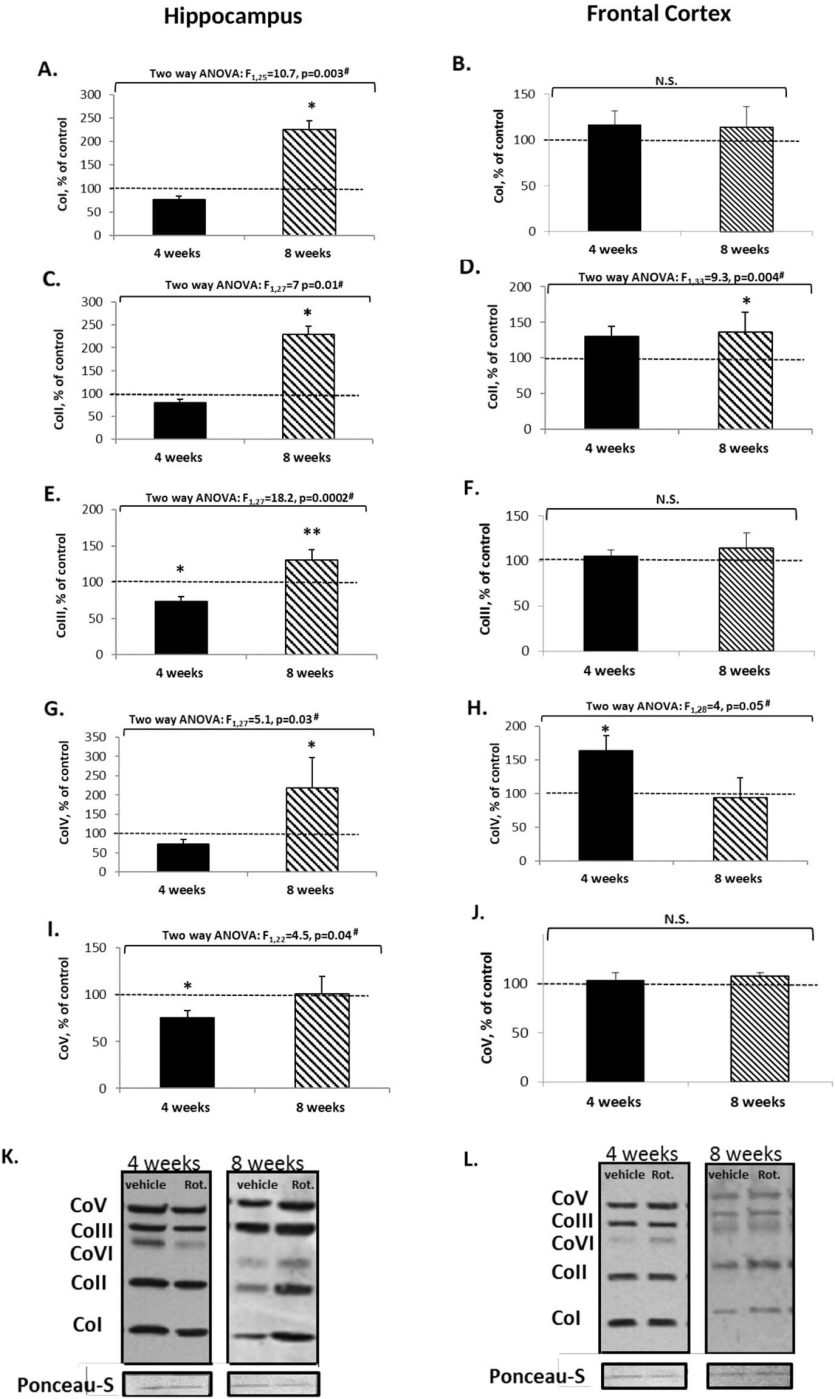
Protein levels of hippocampal (A, C, E, G, I) and frontal-cortex (B, D, F, H, J) mitochondrial respiration complexes following the administration of 0.75 mg/kg/day rotenone for 4/8 weeks. Results in % of control (vehicle) represent means ± SEM of 8–10 mice/group, each in duplicate. Results were analyzed by two-way ANOVA with treatment (±rotenone) and duration (4/8 weeks) as main factors: ^#^values are of the treatment effects. Control values: **Co I**—hippocampus—8773 ± 1275 a.u.; frontal-cortex—13,500 ± 2400 a.u. **Co II**—hippocampus—6937 ± 1390 a.u.; frontal-cortex—13,916 ± 2590 a.u. **Co III**—hippocampus—11,682 ± 961 a.u.; frontal-cortex 8100 ± 2700 a.u. **Co IV**—hippocampus 13,477 ± 1587 a.u.; frontal-cortex 4334 ± 845 a.u. **Co V**—hippocampus 13,773 ± 703 a.u.; frontal-cortex 29355 ± 1576 a.u. **A** Fisher’s LSD post hoc: *8 weeks rotenone vs. vehicle and vs. 4 weeks of rotenone, *p* < 0.00001. **C** Fisher’s LSD post hoc test: *8 weeks of rotenone vs. vehicle and vs. 4 weeks of rotenone, *p* < 0.007. **D** Fisher’s LSD post hoc test: *8 weeks of rotenone vs. vehicle, *p* = 0.01. **E** Fisher’s LSD post hoc test: *4 weeks of rotenone vs. vehicle and vs. 8 weeks of rotenone, *p* = 0.003; **8 weeks of rotenone vs. vehicle, *p* = 0.007. **G** Fisher’s LSD post hoc test: *8 weeks of rotenone vs. vehicle and vs. 4 weeks of rotenone, *p* ≤ 0.01. **H** Fisher’s LSD post hoc test: *4 weeks of rotenone vs. vehicle and vs. 8 weeks, *p* ≤ 0.004. **I** Fisher’s LSD post hoc test: *4 weeks of Rot. vs. vehicle and vs. 8 weeks of rotenone, *p* ≤ 0.00004. **B**, **F**, **J**. N.S. **K**, **L**. Representative blots of complexes I–V (**K**—hippocampus; **L**—frontal-cortex) and Ponceau staining (total protein, for normalization).

We next studied whether the behavioral results are related to brain physiological/biochemical changes. Four weeks of exposure to rotenone resulted in no change in hippocampal basal mitochondrial-respiration (Fig. 4C) but significantly decreased CoIII and CoV proteins levels (Fig. 5E, I) and significantly increased frontal-cortex basal mitochondrial-respiration (Fig. 4D) and CoIV protein levels (Fig. 5H). It might be that the lack of change in hippocampal basal respiration following 4 weeks of rotenone treatment (Fig. 4C) unlike the results in the SH-SY5Y cells and in the frontal-cortex following 72 h and 4 weeks of rotenone, respectively, could be due to the significant decrease in hippocampal CoIII and CoV protein levels (Fig. 5). Elevated frontal-cortex mitochondrial-respiration might be attributable to the increase in CoIV (Fig. 5), indicative of elevated mitochondrial abundance^29^. In parallel with the behavioral tests, the pattern of biochemical changes following 8 weeks of rotenone differed from those revealed after 4 weeks. Significantly decreased hippocampal basal mitochondrial-respiration (Fig. 4C) and increased complexes I–IV protein levels (Fig. 5A, C, E, G, H, K) might reflect attempts to compensate for the distress. Surprisingly, in the frontal-cortex, there were no changes besides an increase in CoII protein levels (Fig. 5B, D, F, H, J, I).

## Discussion

It has recently been suggested that an improved model to study multifactorial disorders such as a psychiatric disorder would be one that resembles the progress of the illness, i.e., mild but prolonged induction of the pathophysiology assumed to be involved^35^. The model is expected to exhibit a disorder phenotype only following the chronic intervention and when additional risk-factors occur. Here, we aimed to create such a model based on mild MD robustly reported in psychiatric disorders, in general, and in BD, in particular^6,36,37^. To this end we focused on chronic exposure to low rotenone or 3-NP doses, expecting to allocate conditions that will mimic the complexity of the disease—fluctuations between manic, depressive and euthymic states. This corroborates the concept that BD involves a gradual decrease in MF^37^, with symptoms beginning only when a threshold is reached or when an event occurs which requires fully functional cells, making a subject more vulnerable to environmental factors targeting MF^38^.

### The cellular (SH-SY5Y cells) model induced by rotenone

To induce gradually aggravated MF leading to accumulation of cellular dysfunction we sought rotenone and 3-NP chronic regimes that do not impair CV but do cause other types of malfunction. Following exposure to rotenone, as robustly reported by others^37,39–42^ we found significantly reduced CV after ≥24 h and 10^3^–10^5^ pM and increased apoptosis following 100 nM for 24 h. Forty-eight hours of exposure to 10^3^–10^5^ pM resulted in significantly reduced basal and ATP-linked OCR paralleling the significant decrease in CV, resembling Yuyun et al.’s report^43^, but differing from others showing a significant reduction in most mitochondrial parameters following only two hrs of 1 nM rotenone^40^. Compatible with Yuyun et al.^43^ we observed a significant increase in MM and ROS-levels following 48 h of 1 and 10 nM and a significant decrease in ΔψM (depolarization) following 10 nM for 48 h.

The exceptionally low rotenone concentration (10 pM) exerted unique effects on SH-SY5Y cells, causing a significant increase in maximal OCR capacity following 24 h of exposure *vs*. a significant decrease in basal and ATP-linked OCR following 48 h, possibly attributable to rotenone being a non-competitive inhibitor of CoI^44^. Namely, at the low concentrationXshort exposure its effect is limited to respiration alterations with mitochondria/cells capable of overcoming its toxic effects, while failing to do so following longer exposure periods. Indeed, 10 pM rotenone did not affect other parameters— CV, depolarization, ROS-levels, and apoptosis. It is worth noting that Yuyun et al.^43^ observed increased basal OCR following 24 h of 12.5–25.0 nM rotenone, accompanied by increased ROS-levels, suggesting a state of mitohormesis, a process wherein low ROS-levels signal initiation of a cascade of cellular events protecting cells from harmful effects, thereby reducing susceptibility to disease^45,46^. Alterations in ΔψM^47^, protein misfolding^48^ and decreased ETC activity^48^, all triggering increased mitochondrial biogenesis, metabolic alterations, increased antioxidant defense and augmented protein chaperone expression, are also suspected to trigger mitohormesis^45^. The observed significant decrease in basal OCR following 48 h of 10 pM rotenone might have triggered the increase in basal and ATP-linked OCR observed following 72 h. Overall, our observed fluctuations in mitochondrial-respiration parameters in response to various durations and concentrations of rotenone may be summarized as follows: in SH-SY5Y cells, mitochondria are capable of overcoming the challenge of <10^3^ pM rotenone for **<**48 h or of 10 pM for 72 h by employing a compensatory effect until its collapse. Failure to cope is then reflected in decreased CV and increased apoptosis following 24 h of exposure to 10^5^ pM rotenone and a decrease in all OCR parameters and ATP levels following 96 h of exposure to 10 pM rotenone. In contrast, as hypothesized, 72 h of 10 pM led to significantly increased basal OCR levels attributable to the increase in CoI levels and activity—a compensatory effect/mitohormesis.

In contrast with the elevated OCR parameters following exposure to 10 pM for 72 h, the decrease in these parameters following 96 h corroborates the significant decrease in CoI activity and the lack of effect on CoI levels. This suggests that the cells/mitochondria, unlike following 72 h, were unable to overcome rotenone’s-induced harm following longer exposure.

#### For the discussion of the effects of 3-NP please see Supplementary information, Discussion

Out of rotenone and 3-NP only rotenone (chronic very low dose) exerted the quested mild mitochondrial-respiration dysfunction, posing the questions whether other mitochondrial-respiration inhibitors may cause similar effects and whether it is unique for CoI inhibitors or exclusive to rotenone. Answering this pondering could lead to further insights into pathophysiology related to minor/mild mitochondrial malfunction and its role in psychiatric disorders, in general, and in BD, in particular.

### The animal (mice) model

As mentioned above, MD has been repeatedly reported in bipolar patients^36,37^. To complement our in vitro model of chronic low rotenone doses, we aimed to establish an in vivo one. We scanned effects of low rotenone doses administered to male mice for different durations. Unlike in the rotenone-induced Parkinson’s-like models employing chronic administration of 2–30 mg/kg/day^19^, we screened a concentration range starting from about one order of magnitude lower (0.25–1.5 mg/kg/day) for 2–8 weeks to induce behavior associated with BD. Similarly to Yu et al.’s finding in aged rats treated with rotenone only (0.5 mg/kg/day for 45 days)^49^, and differently from inducing Parkinson’s-like models by other CoI inhibitors^50^, we show no effect of chronic low rotenone doses on male mice’ spontaneous and motor activity. However, the treatment did induce behavioral changes modeling facets of BD-like characteristics in a dose- and duration-dependent manner. By-and-large, 0.75 mg/kg/day rotenoneinduced duration-dependent dual effects culminating in mania- *vs*. depression-associated behavior following 4 and 8 weeks of treatment, respectively. Remarkably, the representative mania- and depression-associated behaviors in the EPM, FST, and amphetamine-induced hyperlocomotion could be reversed by lithium treatment. Interestingly, in the EPM, lithium by itself affected the behavior in a similar manner to rotenone only. Namely, despite being administrated for 2 weeks both in the 4 weeks and 8 weeks of exposure to rotenone, it increased the time spent in the open arms in the 4-week experiment and decreased it in the 8-week experiment. The increase is compatible with lithium’s anxiolytic effect^51^. The opposite effect of lithium in the 8-week experiment is unexpected. It might have been caused by the difference in the control values between the two experiments. In the FST, unexpectedly (being a well-established both anti-manic and anti-depressive drug), lithium by itself did not affect the behavior, but, as hypothesized, reversed both the mania- and the depression-associated effects of rotenone. In the amphetamine-induced hyperlocomotion, lithium by itself induced its expected anti-manic-like effect both following 4 and 8 weeks of rotenone treatment. Thus, our novel BD-like model contemplates construct, face and predictive validity. To the best of our knowledge this is the first study employing rotenone doses as low as 0.75 mg/kg/day or less for up to 8 weeks. Our reported behavioral changes in response to higher (up to 1.5 mg/kg/day) rotenone doses for 8 weeks corroborate depression-associated behavior observed by others when using high doses of different CoI inhibitors^50^. We discussed above the possible processes mediating the fluctuations observed in the various parameters related to mitochondrial function in the neuronal cells. In a similar manner it is conceivable to speculate that following the relatively short (4 weeks) period of very mild rotenone treatment the mice cope successfully with the respiratory distress in an overshooting manner resulting in ‘over-energetic’ behavior, while they fail to do so following the long (8 weeks) period of rotenone treatment resulting in ‘hypo-energetic’ behavioral state. This is reminiscent of Zeschel et al’s report^52^. The authors conducted a semi-structured retrospective interview for mood swings of patients within eight years of BD onset and found significantly pronounced extremely energetic feeling, racing thoughts, physical agitation, over talkativeness and low sleep requirement occurring most frequently prior to the first (hypo)manic episode, whereas depressed mood, reduced vitality, physical exhaustion, tiredness, and insomnia preceded pre-depressively.

Another BD-like model has recently been described, based on chronic ouabain treatment^53^. We contend that Valvassori et al.’s claimed construct and face validity is weaker than ours: decreased Na^+^/K^+^-ATPase activity *vs*. MD in BD (constract); dichotomic use of tests for either mania- or depression-associated behaviors *vs*. demonstrating both behavioral poles in the same tests (face). Nevertheless, it is attention-grabbing that in a similar manner to ours, the ouabain-induced model also exhibits treatment duration-dependent mania- (after 7 days) and depression (after 14 days)-associated behavior.

In parallel with the behavioral effects, biochemical changes induced by 0.75 mg/kg/day rotenone also demonstrated a time-dependent manner. Increased frontal-cortex but not hippocampal basal mitochondrial-respiration following 4 weeks of exposure might reflect a compensatory response to the challenged mitochondria, corroborating imaging reports of changes in the frontal-cortex of BD patients, in general, and during manic episodes, in particular^54,55^. The reduction in hippocampal basal mitochondrial-respiration following 8 weeks of rotenone parallels brain imaging and reports of decreased volume and functionality in bipolar depression and unipolar depressed patients^54,56–58^.

It is not surprising that the hippocampus and the frontal-cortex exhibited different mitochondrial-respiration parameters response given the different behaviors they mediate^6^. Increased frontal-cortex CoIV protein levels along with increased basal mitochondrial-respiration following 4 weeks of treatment and decreased hippocampal basal mitochondrial-respiration following 8 weeks of exposure parallel the response of SH-SY5Y cells to 10 pM of the drug following 72 and 96 h of exposure, respectively.

Our observed significantly reduced CoIII and CoV or a trend towards reduced (CoI, CoII and CoIV) hippocampal protein levels in parallel with the mania-associated behavior induced by rotenone following 4 weeks of treatment, are compatible with the reported decrease in mRNA levels of some mitochondrial complexes in postmortem bipolar patients’ hippocampus^36^. Intriguingly, elevated hippocampal respiration complexes’ protein levels accompanied the depression-associated phenotype. Although counterintuitive, these results corroborate those reported employing another mania-associated behavioral model. Chronic administration of methamphetamine resulted in significantly reduced brain activity of the mitochondrial-respiration complexes^59,60^. An additional apparent conundrum is lack of concordance between reduced hippocampal basal mitochondrial-respiration but increased protein levels of the respiration complexes following 8 weeks of treatment (the depression-associated phase). Nevertheless, corroborating with the latter, Beech et al. reported upregulated peripheral blood complexes I and III-V mRNA levels in bipolar depressed patients^61^. It is possible that the increase in the levels of the complexes reflects a compensatory effort to overcome the decrease in CoI activity that is insufficient to increase basal respiration.

Prior to summation it should be noted that in a similar manner to the previous attempts to model manic-like or BD-associated behavior in rodents^53,59,60^ our study also used only male mice. In view of the current understanding that there are profound differences between the two genders in multiple aspects^62,63^, future studies should be designed to find out whether the male models are reiterated in female rodents.

To sum-up, here we present an integrative study of in vitro (in cells) and in vivo (in mice) experiments aiming to establish a novel model of mild MD as massively reported in BD pathophysiology. Indices that can be assessed both in cells in culture and in mice are presented for both paradigms with, by-and-large, parallel findings in the cells following 72 and 96 h of exposure to rotenone and in the mice following 4 and 8 weeks of exposure, respectively. Our in vitro model contributes to the understanding of MF and its regulation, e.g., mitohormesis, and supports our hypothesis that very mild respiratory distress induced by 10 pM rotenone for 72 h upregulates mitochondrial respiratory metabolic processes, while exposure to the same dose for 96 h results in neuronal malfunction. The increase in mitochondrial-respiration, CoI and CoV protein levels and CoI activity following 72 h *vs*. decreased mitochondrial-respiration, CoI and ATP levels following 96 h corroborate accumulating evidence indicating increased mitochondrial-respiration and ATP levels in bipolar mania, *vs*. decreased MF in patients in the depressive phase of the illness^35^. Our in vivo model, demonstrating construct, face, and predictive validity, supports the notion that very mild to mild MD is a fundamental player in the etiology of BD. Impaired MF following rotenone treatment was sufficient to induce extreme fluctuations in BD-like behavioral facets, accompanied by cellular and biochemical changes reminiscent of those reported in bipolar patients. Obviously, the depression- and mania-associated behavior may be as well related to other psychiatric disorders. However, finding the same treatment inducing both poles of behavior strengthens the BD-like characteristics in particular. We nevertheless acknowledge that the use of male mice only rather than both female and male mice imposes a lack of generalizability of the findings. Low doses of additional mitochondrial-respiration inhibitors, of CoI in particular, should be tested to confirm or reject this supposition.

## Supplementary information

Supplementary material
