# Supplementary material for: Using mitochondrial respiration inhibitors to design a novel model of bipolar disorder-like phenotype with construct, face and predictive validity
Source: Transl Psychiatry. 2021 Feb 12;11:123. doi: 10.1038/s41398-021-01215-y (PMC7881114; doi:10.1038/s41398-021-01215-y)
Supplement: Supplementary file 1 — Supplementary material [file 41398_2021_1215_MOESM1_ESM.pdf]

Damri *et al - Supplementary material*

I. Supplementary methods

Table I: Antibodies used for Western blotting of mitochondrial respiration complexes

<table>
<tr><th></th><th>Antibody</th><th>Subunit of complexes</th><th>Company</th><th>Dilution[1]</th></tr>
<tr><td rowspan="5">Primary</td><td rowspan="5">total OXPHOS cocktail</td><td>CoI - NADH dehydrogenase [ubiquinone] 1 beta subcomplex subunit 8</td><td rowspan="5">Abcam, Cambridge, UK</td><td rowspan="5">1:1500</td></tr>
<tr><td>CoII – Succinate dehydrogenase [ubiquinone] iron-sulfur subunit II</td></tr>
<tr><td>CoIII - Core protein 2</td></tr>
<tr><td>CoIV - subunit I</td></tr>
<tr><td>CoV - alpha subunit</td></tr>
<tr><td>Secondary</td><td>goat-anti rabbit</td><td></td><td>Santa Cruz Biotechnology, Dallas, Texas</td><td>1:10000</td></tr>
</table>

[1]Primary and secondary antibodies were diluted in Tris Buffered Saline (TBS) with Tween 20 (TBST, Bio-Rad Laboratories, Hercules, CA).

<u>Lithium administration</u>

Eight groups of ICR mice, four injected for four weeks and four – for eight weeks. Among the four weeks-injected mice two received single daily vehicle injections and either regular food (RF) (control) or lithium-supplemented food during the last two weeks of the injections (RF with 0.2% LiCl for five days followed by RF with 0.4% LiCl for additional 10 days[1]) and the other two received, single daily rotenone injections (0.75 mg/kg/day) and either RF or lithium-supplemented food during the last two weeks of the injections. The other four groups received the same regimes except for being injected with either vehicle or rotenone for eight weeks, with lithium administered as above. Lithium plasma levels of the mice were measured in an ion-selective electrode apparatus ISE (AVL 9180 Electrolyte Analyzer, Hoffmann-La Roche, Basel, Switzerland). Lithium blood levels were in the range of 0.56-0.9 mM.

II. **Results** - the effects of 3-NP

The results using 3-NP (Supplementary Fig 1ci-civ) demonstrated a different pattern than those using rotenone as follows. Six hrs of treatment with 10^3 nM resulted in an increase in ROS levels while longer periods did not; mitochondrial mass was gradually decreased with the increase in 3NP dose. However, 24 hrs of exposure to 3-NP did not affect ROS levels but did result in cell and mitochondrial deregulation, namely, in significantly reduced cell viability (Supplementary Fig. 1ci) and mitochondrial mass (Supplementary Fig. 1ciii). It seems that during 24-48 hrs of exposure to 3-NP the reduction in cell viability was not accompanied with elevation in ROS levels.

III. Supplementary figures

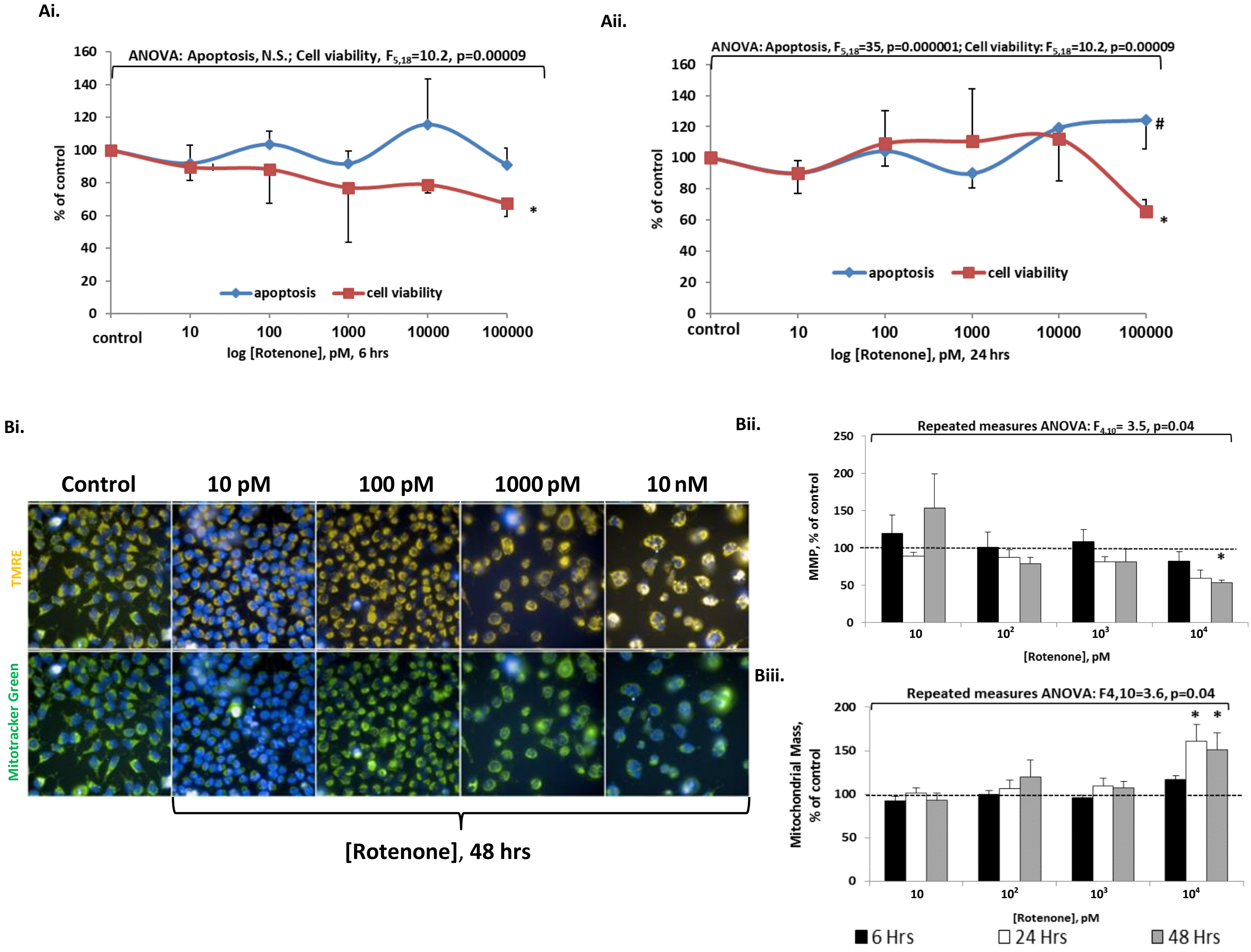

Ciii. Civ.

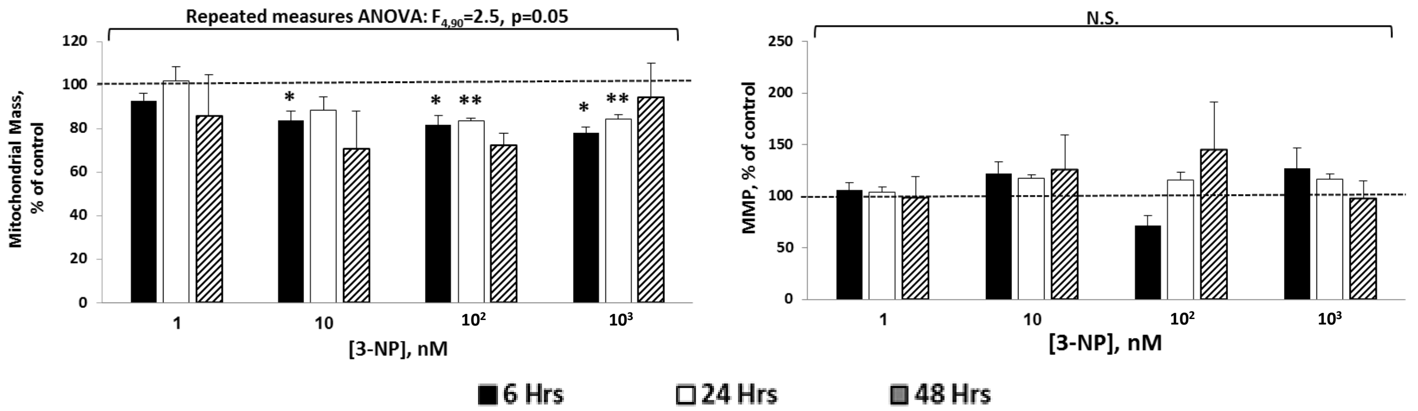

Ci. Cii.

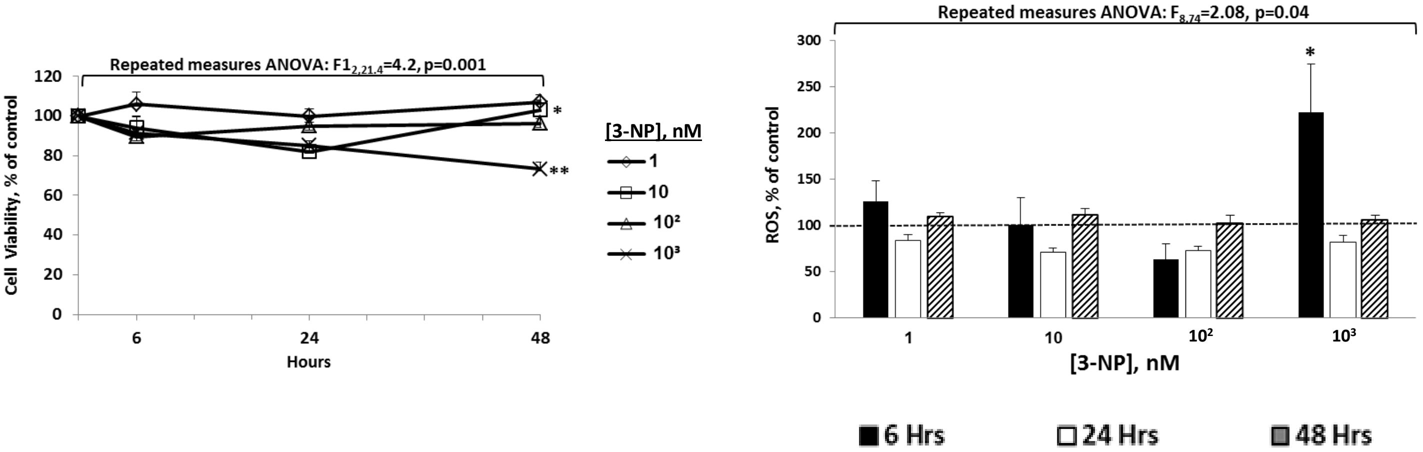

Supplementary Figure 1: Dose-response and time-dependence of the effect of rotenone and of 3-NP on cellular and mitochondrial parameters.

A. Rotenone's effect on cell viability and apoptosis (ApoLive-Glo multiplex assay - sequential determination in the same well). Results are means±SEM of four independent experiments, each in duplicate. Control values were: fluorescence at $400_{Ex}/505_{Em}$ for cell viability - 1418±120 relative fluorescence units (RFU); luminescence at 1 sec for apoptosis - 66246±2673 RLU. **Ai.** CV - 6 hrs, a significant decrease only in cell viability, Fisher's LSD post-hoc test: *10^3 and 10^4 pM rotenone differed significantly from the control, p< 0.05; 10^5 pM *vs.* control, 10 and 10^2 pM rotenone - p<0.003. **Aii.** 24 hrs, 10^5 pM rotenone induced a significant increase in apoptosis and decrease in cell viability. #Apoptosis: Fisher's LSD post-hoc test: 10^5 pM rotenone differed significantly from control, p< 0.05; 10^5 pM *vs.* 10 pM rotenone - p<0.006. *Cell viability: Fisher's LSD post-hoc test: 10^5 pM *vs.* all treatments, p< 0.02. **B. Rotenone's effect on mitochondrial membrane potential (MMP, bi&bii) and mitochondrial mass (MM, Bi&Biii).** Results of three experiments, each in triplicate (>30 cells analysed/well) ± SEM. Control values were: mitochondrial membrane potential – 6492±379 a.u.; mitochondrial mass – 1453±114 arbitrary units (a.u.); **Bi.** Representative images of SH-SY5Y cells incubated with rotenone for 48 hrs and stained with TMRE and Mitotracker green for MMP and mitochondrial mass, respectively. **Bii.** Mitochondrial Membrane Potential Fisher's LSD post-hoc test: *exposure for 48 hrs to 10 nM rotenone *vs.* control and *vs.* 10 pM rotenone for 48 hrs, p<0.07; **Biii.** Mitochondrial mass a gradual increase in mitochondrial mass as a function of rotenone concentration is seen only following exposure for 48 hrs. Fisher's LSD post-hoc test: *exposure for 24 and 48 hrs to 10 nM rotenone *vs.* control, 10, 100 and 1000 pM rotenone for 6 and 24 hrs and to 10 and 1000 pM rotenone for 48 hrs, p<0.04;

C. **The effect of rarely used before 3-NP concentrations (1-10^3 nM) on cell viability (Ci), ROS levels (Cii), mitochondrial mass (Ciii) and mitochondrial membrane potential (Civ).** Results are expressed in percentage of control (vehicle-treated). **Ci.** Cell viability (MTT assay). Control (vehicle-treated cells) values were 0.281±0.032 OD_{540nm}, and are means of three experiments, each in triplicate. Fisher's LSD post-hoc test: 10 nM 3-NP for 24 hrs - p<0.002; 10^3 nM 3-NP for 24 and 48 hrs - p<0.005 and p<0.0004, respectively. cii-civ: Results of fluorescence intensity analysed are means of three experiments, each in triplicate (>30 cells analyzed/well) ±SEM. Control values were: ROS levels – 235.8±27 a.u.; mitochondrial mass – 3734±164 arbitrary units (a.u.); MMP – 3734±164 a.u.; **Cii.** ROS levels Fisher's LSD post-hoc test: 6 hrs, 10^3 nM 3-NP *vs.* all, p≤0.008. **Ciii.** Mitochondrial mass Fisher's LSD post-hoc test did not reveal significant differences between relevant points**.** However, when ANOVA was carried out for each treatment duration separately the results were: *6 hrs, $F_{4,99}$=9.6, p=1E^{-06}; post hoc Fisher's LSD test, 10-10^3 nM 3-NP *vs.* control, p≤0.0005; **24 hrs , $F_{4,90}$=4.4, p=0.002; post hoc Fisher's LSD test, 10^2 and 10^3 nM *vs.* control, p≤0.02; 48 hrs, N.S. **Civ.** MMP, N.S.

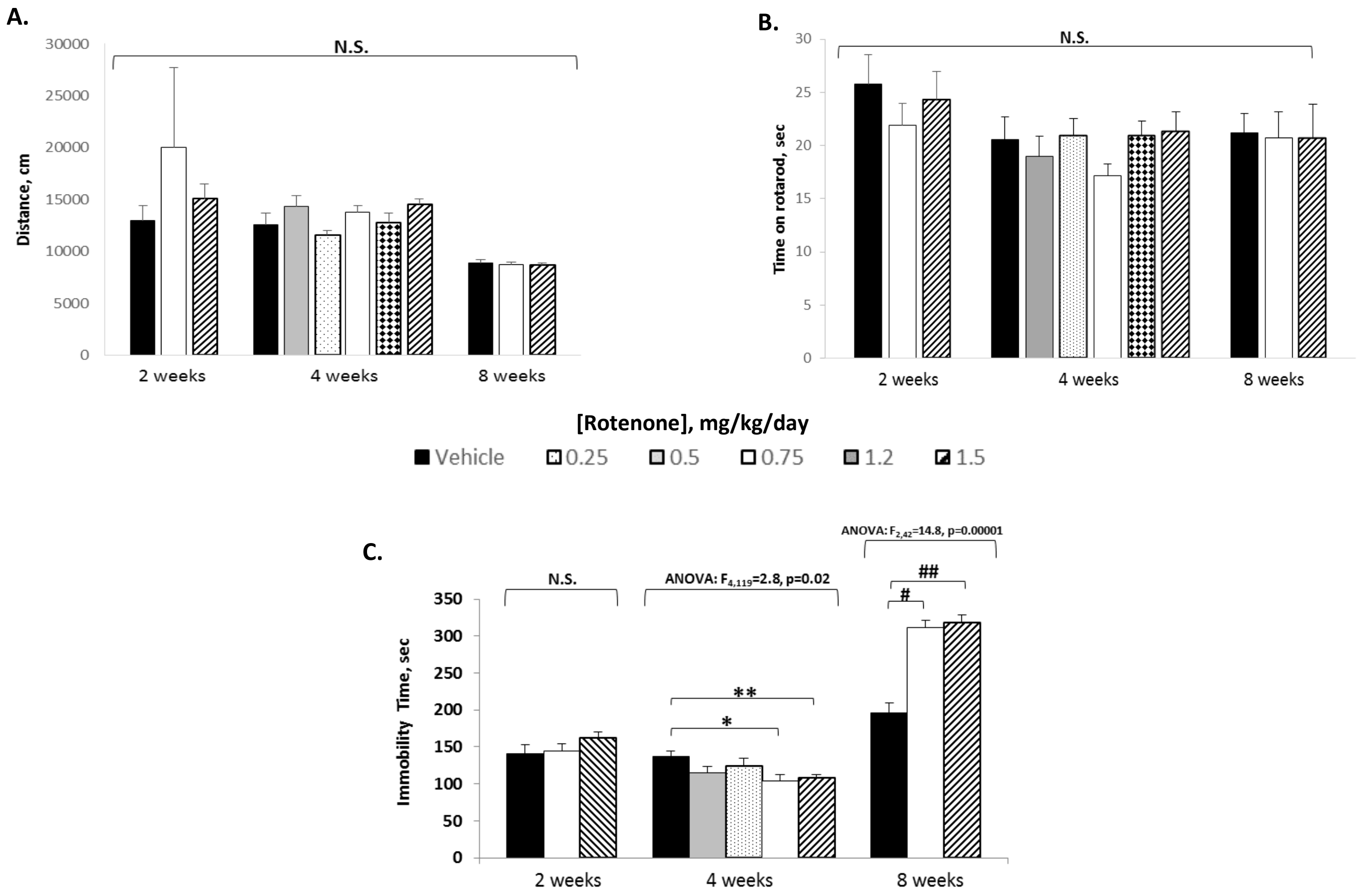

Supplementary Figure 2: Behavioral consequences following two, four and eight weeks of *in vivo* rotenone administration - dose response. Results represent means±SEM of two independent experiments, each with at least 12 mice/group.

A. Spontaneous activity in the open field test – distance traveled.

B. Motor coordination - time stayed on the rotarod.

C. Immobility time in the forced swim test. Four weeks: Fisher's LSD post-hoc test - *0.75 mg/kg/day rotenone *vs*. vehicle, p=0.001; **1.5 mg/kg/day rot. *vs*. vehicle, p=0.05. Eight weeks: Fisher's LSD post-hoc test - $^{\#}$0.75 mg/kg/day rot. *vs*. vehicle, p=0.00002; $^{\#\#}$1.5 mg/kg/day rot. *vs*. vehicle, p=0.00002.

IV. Discussion - the effects of 3-NP

As robustly reported by others[2-4] we found that exposure to 10 nM 3-NP for 24 hrs and to 10^3 nM for ≥ 24 hrs significantly reduced cell viability. We further observed a trend or significant decrease in mitochondrial mass following 6-24 hrs in response to 10-10^3 nM 3-NP. Similar but fiercer findings were observed in a study which tested the temporal and spatial relationship between energy decline, impaired mitochondrial dynamics and neuronal cell death in response to 10 mM (!) 3-NP treatment of cortical neurons for six hours[5]. In this study 3-NP caused an immediate ATP drop, ROS levels rise, profound mitochondrial morphology changes and neuronal cell death. It is conceivable that the higher 3-NP concentration by six orders of magnitude is accountable for the difference between the mild effects on mitochondrial mass in our study as opposed to the extreme damage in Liot et al[5] report. The same contention probably explains why we did not get a significant decrease (depolarization) in $\Delta\psi M$ by 3-NP while other studies which used higher concentrations did find it[6-8]. In our hands, ROS levels were significantly increased following six hrs of exposure to 10^3 nM 3-NP, compatible with others who showed increased ROS levels by exposure to >10 mM 3-NP for <12 hrs[5, 7, 8].

3-NP was reported to enhance autophagy[2, 9, 10]. Others reported interplay between autophagy and ROS levels[11-14]. Hence, it may be suggested that the drastic increase in ROS levels following six hrs of exposure to 10^3 nM 3-NP initiated a cascade of events whereby along with apparently enhanced autophagy, cell viability and mitochondrial mass were decreased (after 24 and 48 hrs for cell viability and after 6 and 24 hrs for mitochondrial mass)[15, 16]. Indeed, excessive autophagy has been shown to result in decreased cell viability, in general[17-19], and following exposure to higher 3-NP levels for different durations, in particular[2, 10].

References

1. O'Brien WT *et al.* Glycogen synthase kinase-3beta haploinsufficiency mimics the behavioral and molecular effects of lithium. *J Neuroscience* 2004; **24**(30)**:** 6791-6798.

2. Solesio ME, Saez-Atienzar S, Jordan J, Galindo MF. 3-Nitropropionic acid induces autophagy by forming mitochondrial permeability transition pores rather than activating the mitochondrial fission pathway. *Br J Pharmacol* 2013; **168**(1)**:** 63-75.

3. Akashiba H, Ikegaya Y, Nishiyama N, Matsuki N. Differential involvement of cell cycle reactivation between striatal and cortical neurons in cell death induced by 3-nitropropionic acid. *J Biol Chem* 2008; **283**(10)**:** 6594-6606.

4. Im AR, Chae SW, Zhang GJ, Lee MY. Neuroprotective effects of Psoralea corylifolia Linn seed extracts on mitochondrial dysfunction induced by 3-nitropropionic acid. *Bmc Complem Altern M* 2014; **14**.

5. Liot G, Bossy B, Lubitz S, Kushnareva Y, Sejbuk N, Bossy-Wetzel E. Complex II inhibition by 3-NP causes mitochondrial fragmentation and neuronal cell death via an NMDA- and ROS-dependent pathway. *Cell Death Differ* 2009; **16**(6)**:** 899-909.

6. Mao Z, Choo YS, Lesort M. Cystamine and cysteamine prevent 3-NP-induced mitochondrial depolarization of Huntington's disease knock-in striatal cells. *Eur J Neurosci* 2006; **23**(7)**:** 1701-1710.

7. Im AR, Chae SW, Zhang GJ, Lee MY. Neuroprotective effects of Psoralea corylifolia Linn seed extracts on mitochondrial dysfunction induced by 3-nitropropionic acid. *BMC Complement Altern Med* 2014; **14:** 370.

8. Colle D *et al.* Succinobucol, a lipid-lowering drug, protects against 3-nitropropionic acid-induced mitochondrial dysfunction and oxidative stress in SH-SY5Y cells via upregulation of glutathione levels and glutamate cysteine ligase activity. *Mol Neurobiol* 2016; **53**(2)**:** 1280-1295.

9. Zhang XD, Qi L, Wu JC, Qin ZH. DRAM1 regulates autophagy flux through lysosomes. *PLoS One* 2013; **8**(5)**:** e63245.

10. Pereira GJ, Tressoldi N, Hirata H, Bincoletto C, Smaili SS. Autophagy as a neuroprotective mechanism against 3-nitropropionic acid-induced murine astrocyte cell death. *Neurochem Res* 2013; **38**(11)**:** 2418-2426.

11. Fang C, Gu L, Smerin D, Mao S, Xiong X. The interrelation between reactive oxygen species and autophagy in neurological disorders. *Oxid Med Cell Longev* 2017; **2017:** 8495160.

12. Kaushal GP, Chandrashekar K, Juncos LA. Molecular interactions between reactive oxygen species and autophagy in kidney disease. *Int J Mol Sci* 2019; **20**(15).

13. Filomeni G, De Zio D, Cecconi F. Oxidative stress and autophagy: the clash between damage and metabolic needs. *Cell Death Differ* 2015; **22**(3)**:** 377-388.

14. Cordani M, Donadelli M, Strippoli R, Bazhin AV, Sanchez-Alvarez M. Interplay between ROS and autophagy in cancer and aging: from molecular mechanisms to novel therapeutic approaches. *Oxid Med Cell Longev* 2019; **2019:** 8794612.

15. Benischke AS *et al.* Activation of mitophagy leads to decline in Mfn2 and loss of mitochondrial mass in Fuchs endothelial corneal dystrophy. *Sci Rep* 2017; **7**(1)**:** 6656.

16. Dalle Pezze P *et al.* Dynamic modelling of pathways to cellular senescence reveals strategies for targeted interventions. *PLoS Comput Biol* 2014; **10**(8)**:** e1003728.

17. Neufeld TP. Autophagy and cell growth--the yin and yang of nutrient responses. *J Cell Sci* 2012; **125**(Pt 10)**:** 2359-2368.

18. Das G, Shravage BV, Baehrecke EH. Regulation and function of autophagy during cell survival and cell death. *Cold Spring Harb Perspect Biol* 2012; **4**(6).

19. Bialik S, Dasari SK, Kimchi A. Autophagy-dependent cell death - where, how and why a cell eats itself to death. *J Cell Sci* 2018; **131**(18).
